# Women's experiences of how their recovery process is promoted after a first myocardial infarction: Implications for cardiac rehabilitation care

**DOI:** 10.3402/qhw.v11.30633

**Published:** 2016-05-10

**Authors:** Inger Wieslander, Jan Mårtensson, Bengt Fridlund, Petra Svedberg

**Affiliations:** 1 School of Social and Health Sciences, Halmstad University, Halmstad, Sweden; 2 School of Health Sciences, Jönköping University, Jönköping, Sweden

**Keywords:** myocardial infarction, promotion, qualitative content analysis, recovery process, women

## Abstract

**Background:**

A rapid improvement in the care of myocardial infarction (MI) in the emergency services has been witnessed in recent years. There is, however, a lack of understanding of the factors involved in a successful recovery process, after the initial stages of emergency care among patients, and in particular those who are women. Both preventive and promotive perspectives should be taken into consideration for facilitating the recovery process of women after a MI.

**Aim:**

To explore how women's recovery processes are promoted after a first MI.

**Methods:**

A qualitative content analysis was used.

**Findings:**

The women's recovery process is a multidirectional process with a desire to develop and approach a new perspective on life. The women's possibility to approach new perspectives on life incorporates how they handle the three dimensions: behaviour, that is, women's acting and engaging in various activities; social, that is, how women receive and give support in their social environment; and psychological, that is, their way of thinking, reflecting, and appreciating life.

**Conclusions:**

The personal recovery of women is a multidirectional process with a desire to develop and approach a new perspective on life. It is important for cardiac rehabilitation nurses to not only focus on lifestyle changes and social support but also on working actively with the women's inner strength in order to promote the recovery of the women.

A rapid improvement in the care of myocardial infarction (MI) in the emergency services has been witnessed in recent years (Stenestrand, Lindahl, & Wallentin, 2007). There is, however, a lack of understanding of the factors involved in a successful recovery process, after the initial stages of emergency care among patients, and in particular those who are women. Coronary heart disease (CHD) is the major cause of death and disease burden among women in the world (Gaziano, Bitton, Anand, Abrahams-Gessel, & Murphy, 2010). A first MI annually affects more than 10,000 women in Sweden and approximately 70% of them survive (The National Board of Health and Welfare in Sweden, 2013). The recovery process for women after discharge from a hospital (Johansson Sundler, 2008), the impact of the MI, and the support from healthcare professionals influence this process (Gregory, Bostock, & Backett-Milburn, 2006; Johansson Sundler, 2008). Little is, however, known about how to best promote the recovery of women after their first MI.

A more promotive approach should be taken into consideration in order to facilitate women's recovery (World Health Organization [WHO], 1986). International and national guidelines and recommendations have developed over time, but these are still primarily focused on the secondary prevention of disease progression and risk factors (Lloyd, 2009; Strid, Lingfors, Fridlund, & Mårtensson, 2012; Swedeheart, 2013). A cardiac rehabilitation programme (CRP) is defined as an important part of secondary prevention after MI (The Swedish National Board of Health and Welfare, 2008) and usually includes education and counselling services to help heart patients increase physical fitness, reduce cardiac symptoms, improve health, and reduce the risk of future heart problems (American Heart Association, 2014). Changing from a focus on prevention to one on factors that can strengthen the individuals’ own health and recovery generates new challenges for nurses in clinical practice (Svedberg, 2011; The Swedish Society of Nursing, 2008; Whitehead, 2006). The concept of recovery has been defined in different ways depending on context, culture, and profession (Jackson et al., 2003; Slade & Hayward, 2007) and is a widely used concept in the psychiatric rehabilitation. However, it is still underdeveloped in other care areas such as cardiac rehabilitation. It has been stated that professionalized clinical models focus on treatment and on improvement regarding symptoms and functions, while recovery models tend to put more emphasis on empowerment and on the person's own experience of health (Mueser et al., 2002; Slade & Hayward, 2007). Thus, it does not only refer to the remission of clinical symptoms and functions; it is also described as being a broader concept that incorporates the person's whole life situation. There is a distinct difference between the concept of recovery and rehabilitation, but these terms have often been used interchangeably in healthcare (Deegan, 1988). Recovery includes both clinical and personal recovery, where the former corresponds to rehabilitation and a professional perspective focus on reducing symptoms and improving functioning (Mancini, Hardiman, & Lawson, 2005; Slade & Hayward, 2007), while personal recovery is seen as a multidimensional ongoing holistic process to health (Anthony, 1993; Deegan, 1996; Mancini et al., 2005; Slade & Hayward, 2007). Therefore, it is not sufficient for healthcare professionals to mostly focus on rehabilitation and the clinical recovery. A greater understanding of the concept of personal recovery among women with MI could thus be an important contribution to the development of CRP.

Very little research has been carried out into factors that can strengthen and contribute to promoting women's personal recovery processes. Research into women's experiences after an MI has had a specific focus on the difficulties in their daily lives and the interruptions and limitations that occur (Johansson, Dahlberg, & Ekebergh, 2003; Sjöström-Strand, Ivarsson, & Sjöberg, 2011; Stevens & Thomas, 2012; Svedlund & Danielson, 2004). Other studies have primarily focused on health care interventions such as CRPs (Day & Batten, 2006; Simony, Dreyer, Pedersen, & Birkelund, 2015) and patient education (Perry & Rosenfeld, 2005) as well as how MI affects patients’ health behaviours (McSweeney & Coon, 2004) and intimate relations (Arenhall, Kristofferzon, Fridlund, Malm, & Nilsson, 2011). Several studies have also shown that fewer women than men actually participate in CRPs (Daniels, Arena, Lavie, & Forman, 2012; Grace, Racco, Chessex, Rivera, & Oh, 2010). Further research into women's needs in the recovery process is thus needed. A meta-synthesis of women's recovery (Hildingh, Fridlund, & Lidell, 2007) concluded that the recovery process is experienced as being complex in terms of having to cope with balancing between being oriented towards themselves and towards others. Recovery is described from the women's own perspective as a gradual and personal ongoing process, which includes the need for changing priorities and learning to adapt to a new life situation (Norekvål et al., 2008; Tobin, 2000) and the need to receive good care (Tobin, 2000). Healthcare professionals have described that women's recovery process depends both on individual factors such as their ability to cope with the stresses in life and on the available support from those around them (Wieslander, Mårtensson, Fridlund, & Svedberg, 2013). There is thus a paucity of research into contributory factors for promoting women's recovery and a lack of understanding as to why CRP remains underutilized among women. The aim of this study was thus to explore how women's recovery process is promoted after a first MI. Such knowledge may help to develop individually based interventions in accordance with the needs of women in their recovery process.

## Methods

### Design and setting

The study had an explorative and descriptive design with an inductive approach in order to gain knowledge and understanding of the investigated phenomenon and used a qualitative content analysis (Downe-Wamboldt, 1992). The interviews were carried out between April 2010 and December 2010 at 10 hospitals (4 university, 3 county, and 3 district hospitals) geographically distributed in southern and central Sweden. The catchment area population was approximately 3 million people out of a total population of 9.6 million in Sweden (SCB, 2013). These three settings include hospitals from both urban and rural areas, thus encompassing the major variations to be found in the Swedish healthcare services (Swedeheart, 2013). All the included hospitals follow the guidelines for Swedish cardiac rehabilitation (The National Board of Health and Welfare in Sweden, 2015) and the CRP includes organized physical exercise and a predetermined educational content.

### Participants

The inclusion criteria were women: suffering a first MI during 2009, participating or not participating in a CRP, and being able to understand, read, and speak the Swedish language. Cardiac rehabilitation nurses (CRNs) at the 10 hospitals asked women who met the inclusion criteria whether they were interested in being further informed about this study. The first author contacted the 28 women, who accepted the invitation, and 26 of these consented to participate and two declined due to time constraints. Agreement was made with each of the 26 women about a time and place for the interview. The women included in the interviews were between 45 and 74 years; one woman was born outside Sweden, 17 lived in an urban environment and 14 participated in a CRP. For socio-demographic characteristics of the sample, see Table I.

**Table I:** Socio-demographic data of women (*n*=26).

Age (mean; range)	(60; 45–74)
Education	
Primary school	8
Secondary school	11
University	7
Living situation	
Single	5
Cohabiting	21
Place of residence	
Urban	17
Rural	9
Participating in cardiac rehabilitation programme	
Yes	14
No	12
Work situation^+^*before a first MI*, ^++^ *after a first MI*	
100%	15^+^ 9^++^
75%	0^+^ 3^++^
50%	3^+^ 3^++^
Unemployed	0^+^ 1^++^
Disability pension/Retired	8^+^ 10^++^

### Data collection

Data was collected in open-ended interviews, which were conducted and recorded by the first author (IW), 13 were performed by telephone and 13 face-to-face in an undisturbed place in the women's homes, at a hospital or the first author's place of work. The first author, an RNIC and familiar with the context, had no previous experience of the practices at the 10 included hospitals. All the interviews were carried out 7–12 months after the women's first MI. The interviews started with an open question “Can you describe what a typical day looks like now after the MI?” followed by the main question “What do you think has promoted your recovery?” This was followed by prompting questions around those things which strengthened and contributed to their recovery. In order to attain greater depth in the data, follow-up questions such as “What do you mean when you say …?” and “Can you explain more about …?” were asked. Two pilot interviews were conducted in order to test the opening questions and these interviews were included as no amendment was required. The audio-taped interviews took the form of a dialogue (Kvale & Brinkmann, 2009), lasting 45–85 min. All the interviews were transcribed verbatim and resulted in 179 double-spaced pages (A4, Font size 12). Saturation was reached after 18 interviews; from this point, the collection of new data did not shed any further light on the phenomenon under investigation.

### Ethical consideration

The Regional Ethical Review Board in Linköping (2007-06-12, Dr. No 104-07) approved the study, which adhered to the requirements of the Declaration of Helsinki (World Medical Association, 2008). Informed consent was obtained after the participants were fully informed, both verbally and in writing, about the aim of the study, the voluntary nature of participation, and the possibility of withdrawing. The heads of the cardiology departments approved the study.

### Data analysis

**Figure 1:**
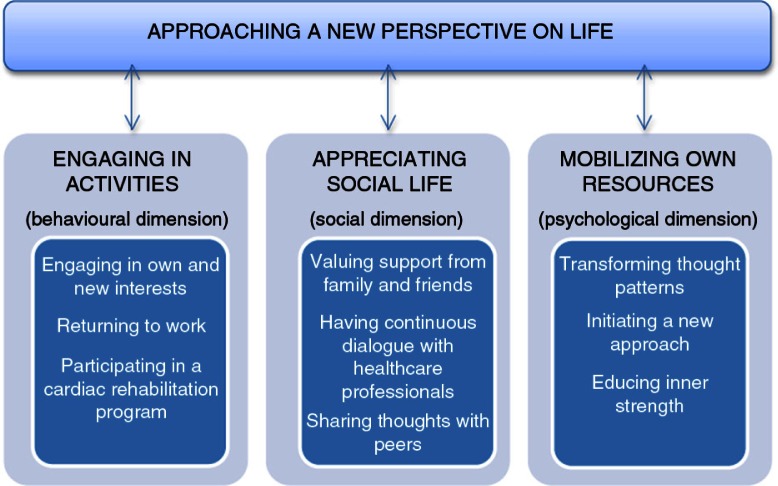
Illustration of the overall theme, categories and related subcategories.

Data were processed by the main author, in collaboration with the co-authors, using qualitative content analysis, where the manifest content (what the text says) and the latent content (the interpreted meaning) were analysed in accordance with Graneheim and Lundman (2004). All authors were nurses and well-established in qualitative methodology and cardiac rehabilitation, both in theory and practice. The interviews were read through several times to gain familiarity, and meaning units related to the aim of the study were placed in an analytical matrix. These were then condensed without losing the core of the content. In the next step, the condensed meaning units were abbreviated to codes that were compared, based on differences and similarities, and nine subcategories were created forming three categories on a manifest level. The three categories based on the content were then abstracted and finally an overall theme was formulated (Figure 1), which expressed the latent meaning of the content (Graneheim & Lundman, 2004). Representative quotations from the women are used to illustrate the data in the categories.

### Rigour

Rigour in the data collection and data analysis was ensured by using the concepts of credibility, dependability, and transferability (Graneheim & Lundman, 2004). Credibility refers to confidence in the truth concerning data collection and analysis. A purposive selection of women with a first MI was chosen in order to strengthen credibility (Graneheim & Lundman, 2004). The data, based on 26 interviews including two pilot interviews, was collected and the authors worked both individually and together during the analysis until a consensus was reached. The chosen quotations reflecting the content of each category provide the reader with an opportunity to determine the credibility of the study (Graneheim & Lundman, 2004). The women were asked by the CRN if they wanted to participate. A weakness may be that the CRNs can have selected the women they considered most suitable for the study. It is possible that other aspects of the phenomenon would have appeared if the recruitment procedure had been performed differently. Another aspect of credibility is the data collection procedure. The women were free to choose if they wanted to be interviewed by telephone or face-to-face. This could constitute a limitation, but there were, however, no differences in the duration of the interviews or in the content and meaning of the analysed data between the two different approaches. The interviews, both by telephone and face-to-face, provided a large amount of data and the variety of socio-demographic characteristics and the different experiences of CRP among the women provided a good possibility for different aspects of the phenomenon under investigation to be highlighted (Graneheim & Lundman, 2004). Dependability can be regarded as reliability (Graneheim & Lundman, 2004) and was strengthened by the fact that the interviews were performed with the same main question and the follow-up questions stimulated the women to reflect and further expand their thoughts regarding the recovery process. Dependability was also strengthened by a continuous dialogue that took place among the authors so that our opinions concerning differences and similarities of content were consistent over time. The results from studies performed with a qualitative design are generally not transferable to the population at large (Graneheim & Lundman, 2004) but can be highly relevant for women with CHD. The results can thus serve as a valuable reference for nurses to better understand women's recovery after an MI. In order to assess if the study can be applied to other settings, we have attempted to describe the participants and context as clearly as possible.

## Findings

The results include the overall theme “Approaching a new perspective on life” and three related categories “Engaging in activities,” “Appreciating social life,” and “Mobilizing own resources” that describe women's recovery process after a first MI.

### Approaching a new perspective on life

In women's recovery process, they are approaching a new perspective on life, which includes enjoying and valuing good moments in everyday life and not taking life for granted. The recovery process did not just concern their disease, but also their views of existence and a transition to a different life than before. The women's possibility to approach new perspectives on life incorporates how they handle the behavioural, social, and psychological dimensions. The behavioural dimension concerned how the women engaged in both personal and illness-related activities to promote their recovery. The social dimension concerned how the women in their social environment received support from family, friends, peers, and healthcare professionals as well as gave support and shared thoughts with peers in the same situation. The psychological dimension included their transformation in thinking, how they initiated a new approach in life and how they educed their inner strength. The women's ability to embrace these dimensions in their recovery process affected their sense of confidence and belief in a long life.

### Engaging in activities

The category “engaging in activities” comprised a behavioural dimension that involved *engaging in own and new interests*, *returning to work* and *participating in a cardiac rehabilitation program* in order to promote their recovery.

*Engaging in own and new interests* concerned how the women in their everyday lives after an MI prioritized engaging in both previously established as well as new interests, which was essential for their recovery process. These interests included singing in a choir, reading books, solving crosswords, sewing and needlework as well as physical exercise in different forms. Their engagement in new interests is an indication of the women prioritizing life differently after their MI; these activities provided them with a distraction from difficult thoughts as well as giving them pleasure and well-being in everyday life. “I really like gardening and I have noticed that it makes me feel better” (n3).

Those who enjoyed travelling had continued to do so in order to see, learn, gain new experiences, and meet new people. This was not only to develop a greater interest in activities they previously had but also to engage in new interests and new activities, thus gaining new circles of friends “… and the tango course I attended, the joy, because I started to dance, it was a try-it-out course/an introductory course, so I started and as a result gained new friends” (n5). Exercise that contributed to their recovery included cycling, walking long distances, exercising regularly in a gym, swimming several times per week as well as gardening, riding, yoga, and dancing. “I love to go out for long walks and I prefer to go alone”(n4). They exercised more regularly after the MI and realised that these types of activities created a sense of well-being.

Another factor that was highlighted as contributing to their recovery was *returning to work*. The women talked of returning to work as being important in order to distract their thoughts, to meet with colleagues, and to start with an ordinary life again. Some women had changed workplace due to previous conflicts and some decided to work less to have more time at home and to avoid stress. “… then there was a conflict at my former workplace … and I finally said “I don't want to stay here” … my job position was simply moved, so I got a brand new head, and I kept my full-time position …”(n5). Some women received and appreciated the opportunity to take courses that the employer offered.

The women talked of learning more about their health and the importance of physical training by *participating in a cardiac rehabilitation program (CRP)*. When participating in these programmes their recovery was supported by meeting the rehabilitation team; consisting of a cardiologist, a nurse, a physiotherapist, and a social worker, and by accessing their knowledge and experience and the easy-going atmosphere. “… I thought it would be really interesting, because I enjoy learning new things, I've always been interested in health. So I thought that attending the programme was a real bonus …” (n14). The women appreciated the healthcare professionals’ friendly and knowledgeable approach during CRP and that the atmosphere was relaxing, which led to a feeling of security. They liked the structured physical training, where they were instructed to run, jump, and move and did not have to worry about over-exerting themselves during the 1 h of hard training led by a physiotherapist.

I really believe that my life has changed for the better following the Myocardial infarction … well, for example, the heart school taught me a lot and gave me the opportunity to take up gymnastics which did so much for me. Otherwise I wouldn't have dared to pushed myself so much (n23).

The women appreciated the fact that they, if unable to afford it, were able to participate through receiving bus passes from the municipalities. The women said that the participation in CRP supported them in their recovery process.

### Appreciating social life

This category had a social dimension that included the importance of *valuing support from family and friends*, *having continuous dialogue with healthcare professionals* and *sharing thoughts with peers* in order to promote the women's recovery process.

In terms of *valuing support from family and friends* the women spoke of the importance of their relationships with family members and others in everyday life and that this was essential for their recovery. Talking to their partner and their children, experiencing being listened to as well as receiving help with housework generated feelings of support and being treated with respect and feeling intimacy inspired strength.

… Nowadays I make the most of my time. I don't go running back and forth each evening any more. I think it's important to be able to sit down with my husband have a chat over a cup of coffee or a glass of wine or whatever we feel like (n14).

Some stated that they valued their families more than previously and some of their partners attended the CRP, thus gaining information about how the women might feel following an MI, which the women found valuable. Spending time with their grandchildren and being allowed to help out meant a great deal to them. “I have a grandchild who I love to take care of, who I often pick up from preschool when mum and dad can't get away from work” (n20).

The women valued their friends (mainly female) for keeping in contact via telephone, cards or text messages but also for practical advice that they could assimilate. Other contributory factors to their recovery were: being able to share their thoughts and feelings, experiencing closeness with their female friends, getting support from people at work, at the church, from siblings and parents, and spending long periods in their holiday home together with family and friends. “… then I got help and support from friends, but also from my family and then from work … yes that's what it was like … I really felt it” (n2).

*Having continuous dialogue with healthcare professionals* was important for the promotion of their recovery process. The women experienced that their new situation was stressful and new concerns arose with time, and thus a continuous dialogue with healthcare professionals was essential. The women valued that the healthcare professionals saw and listened to them as persons and did not just focus on the MI diagnosis. The women valued easy accessibility and that the professionals answered their questions, which made them feel they were taken seriously and that their concerns were listened to.

… and then you were assigned a nurse who you could turn to when you were at home … it helped me a lot … if you felt that things were tough then you could call her, and there lots of times that helped (n22).

Through the dialogue the women perceived that they were supported and encouraged to recover. Moreover, they also appreciated the information and consultation with healthcare professionals about prescribed medication and side effects. The combination of the warm atmosphere, knowledge, and attentiveness provided by the healthcare professionals created a sense of security as well as an inspiration to work with their recovery process. “I received a lot of help from everybody, really … the cardiac rehabilitation nurses … As I can't stand stressful situations” (n14).

*Sharing thoughts with peers* contributed to the women's recovery through encountering others in the same situation in the CRP. “… the whole little group had the same diagnosis. That was positive. We really had a lot to talk about” (n19). The women shared experiences, encouraged, and stimulated each other as well as joking together, which provided a sense of self-confidence and well-being.

… and it's very much about meeting other people in the same situation. We sort of pep each other a little. The atmosphere is quite good, everybody is in a cheerful mood. You never laugh at but with somebody and that's a great difference. It also makes it less difficult for the person (n1).

### Mobilizing own resources

This category has a psychological dimension in which it was described how the women mobilized their own resources in order to promote their recovery by *transforming thought patterns*, *initiating a new approach*, and through *educing inner strength*.

The women spoke of needing to work actively with *transforming thought patterns* during their recovery process in order to change their mindset concerning their lives. Transforming thought patterns involves daring to assimilate new knowledge and to think differently in many situations in order to manage the stress in their lives.

I'm kinder to myself today, because earlier I used to think I was stupid for not understanding many things … (n5).

They described it as if they dared to be open to changing how they were thinking; thus contributing to new understandings and new explanatory models of life. When the women had started to re-evaluate their own thought patterns, they also managed to say no to other people in different situations and not to please everyone else in an effort to obtain approval. Attempting to reflect more about their own lifestyle and realizing that some things may take more or less time to work through also facilitated their recovery.

I've always been like, if anyone says ‘I wonder where it is’ – Yes, I know! I can get it!” Now I'm learning to sit still, nobody says that you must do everything. And I realise that saying no is not a matter of life and death. But it's strange, it's always been that I have to feel appreciated, be told that I'm good (n3).

*Initiating a new approach* concerned the women making new priorities in life and starting to change their behaviour with regard to food, smoking, work, and education. By wanting to start a new approach in life, they invested time and energy to reduce overweight through combining exercise with a new diet. The goal for this investment was to gain a normal blood sugar level. They also invested time in other different lifestyle changes such as giving up smoking. Many of them chose to reduce their working hours and evening activities as well as commitments outside the home, because they wanted more time for the activities they themselves had chosen and which gave them pleasure in life.

I have one whole day off during the week so that's changed a great deal. My employers were really happy to agree to that (n14).

They considered how to proceed with their lives by for example continuing a previously interrupted education or starting a new company. They also tried to cope with worry in a different way and did not allow it to dominate. “I'm 14 kg lighter and there's a long way to go but it's still 14 kg. At least it's in the right direction” (n20).

In *educing inner strength*, the women struggled to give themselves first priority after having previously more or less deferred their own needs and helped others instead.

Today, sometimes I don't bother dusting in the corners. I have slowed down. I say “No, thank you” to some things, like taking an active part in different associations and boards, I still have some positions left, but I have chosen which ones (n19).

Their recovery process was facilitated by having a positive attitude to life, trying to make the best of a situation and focusing on opportunities instead of obstacles as well as being determined to look ahead. They educed their inner strength in order to do new things, gained a new approach and broadened their horizons by making small changes in everyday life. “I really think my everyday life is almost priceless … so calm and I own my time and can do whatever I want” (n11). They tried to be kinder to themselves, learned to say no and not end up as always being there for others and being “a good girl.” “I feel now that, in my condition, I have the right to care more about myself” (n4). They felt that they had the right to consider themselves more and thus let go of a number of musts, such as cleaning and duties for various associations.

The children felt a bit like … that I shouldn't do so much because I'm the kind of mother who has a guilty conscience about it because I feel … that it's better for me to rest, sit down and read a book or just feel good … it has meant a lot to me (n14).

## Discussion

This result highlights the need to take behavioural, social, and psychological dimensions into account in CRP in order to promote women's recovery process. Their personal recovery is a multidirectional process, which means that the process is not linear but instead operating in several directions at the same time. The intention is to develop an approach to a new perspective on life, which does not entail returning to the previous life situation. Traditionally, CRP is performed in peer groups with predetermined education material regarding MI and organized physical exercise (Perk et al., 2012; Piepoli et al., 2010; WHO, 1993) and is based on the purpose of secondary prevention: 1) preventing disease progression, 2) reducing death by focusing on lifestyle factors, and 3) medical treatment of risk factors (The National Board of Health and Welfare in Sweden, 2008). From the findings in our study, nurses ought to work with a more recovery-oriented holistic approach (Davidson, 2008; WHO, 1986) where behavioural, social, and psychological dimensions are taken into account. This is in line with a recent study (Simony et al., 2015) that showed that recovery after an MI, for both women and men, is a matter of finding new values in lives through focusing on individual needs and subjective well-being.

A further contribution from our study is the behavioural dimension in which the women emphasized the importance of being engaged in different activities in order to promote their recovery process. They talked of the importance of participating in CRP and returning to work, which has been described in earlier research (Sjöström-Strand et al., 2011; Stevens & Thomas, 2012). Another aspect of the behavioural dimension, which was essential for their recovery process, was the re-evaluation of the activities they usually performed, and to engage in new meaningful activities such as singing in a choir, reading books and travelling. These new interests are an indication of women prioritizing life differently after their MI. Re-evaluating and having the ability to change lifestyle activities among women post-MI, have shown to improve their inner strength and self-esteem (Mendes, Roux, & Ridosh, 2010).

A meaningful part of their recovery process was the psychological dimension that incorporates how they use their inner strength and mobilize their way of thinking in order to increase their ability to cope with the stresses of life and to increase their well-being. Earlier research indicates that women with MI experience a great deal of worry and anxiety, especially during the first 4 months (Alsén, Brink, Persson, Brändström, & Karlson, 2010; Kerr & Fothergill-Bourbonnais, 2002; Sjöström-Strand et al., 2011; Svedlund, Danielson, & Norberg, 2001; Tobin, 2000). It is thus essential that the CRN has the possibility to discuss concerns and ways of thinking with the women so that the stress does not become pathological and interferes with their ability to recover. CRNs highlight that the need for support differs in length of time for women after an MI (Wieslander et al., 2013). Women have a lower level of sense of coherence and health status scores at the beginning of their recovery and 12 months later compared to men (Bergman, Årestedt, Fridlund, Karlsson, & Malm, 2012; Dreyer et al., 2015). Thus, it is important for the healthcare services not to put a time limit for CRP or for individualized support from CRNs, because there could be critical aspects and events that appear later in the recovery process that can affect the women's well-being. A longer CRP with extended support could improve women's chances of recovery. Patients, both women and men, describe that the supportive care is too limited, because it is sometimes hard to participate in every meeting during the CRP (Simony et al., 2015). Since CRP usually have a predetermined time frame and follow a set structure that is similar for all patients, this could entail that women are unnecessarily passive in their personal recovery. It is important for CRN not to underestimate the women's inner resources and the ability to be active in their own care.

A common and essential dimension in women's recovery is their relationships with other people (social dimension). The women in this study value the support from family and friends, as well as sharing thoughts with other women in the same situation during their recovery process, which is confirmed in other studies (Doiron-Maillet & Meagher-Stewart, 2003; Mendes et al., 2010; Pryor, Page, Patsamanis, & Jolly, 2014; Svedlund & Danielson, 2004; White, Hunter, & Holttum, 2007; Wieslander, Baigi, Turesson, & Fridlund, 2005) and is evident as a contributing factor for health (Sjöström-Strand & Fridlund, 2006; White et al., 2007). Social support is also a contributory factor for men's recovery (Kristofferzon, Löfmark, & Carlsson, 2003; Pryor et al., 2014). Despite the importance of support for women's recovery, they experience less social support up to 1 year after an MI compared to men (Kristofferzon et al., 2003). The support from healthcare professionals was described as very significant if the support was based on a dialogue with the CRN and when the women were seen as persons and not only as a diagnosis. Women have in previous studies spoken of a lack of personalized support (Doiron-Maillet & Meagher-Stewart, 2003; Johansson et al., 2003; White et al., 2007) leading to uncertainty and a delay in their recovery process. It is, however, of great importance for CRN to not only focus on lifestyle changes and risk factors of MI, but also to work actively with the psychological and social dimensions in order to promote their recovery process. This highlights the need for CRN to base their actions on a recovery-oriented approach with a holistic perspective on women's health (Davidson, 2008; WHO, 1986). Understanding factors that promote the recovery process after an MI from the perspective of the woman gives a basis for CRNs when planning the best possible support for these women. The knowledge may aid the CRN in being alert to mapping the individual needs early and, based on this, plan appropriate activities together with the woman. These activities need to be offered both individually and as CRP. After women's completion of the CRP, it is important that the CRN discusses the need for continued support with the women, this due to the women needing more time to recover.

Finally, it is important to discuss if there are any gender differences after an MI. We know today that women have a poorer prognosis then men (Berg, 2013; Rosengren et al., 2001), women have been less likely to be referred to CRP by healthcare professionals (Daniels et al., 2012; Grace et al., 2010, 2015) and that women tend to not complete CRPs to the same extent as men (Grace et al., 2015). Women after an MI experience lower quality of life, lower health status, and a lower sense of coherence than men do (Bergman et al., 2012; Dreyer et al., 2015; Wrześniewski & Włodarczyk, 2012). Women and men have also described that they have different recovery goals after an MI that seem to follow traditional gender-role patterns (Grande & Romppel, 2011; Kristofferzon et al., 2003). The ability to perform household duties and independence in activities in daily life is described as an important goal for women while resuming work and keeping physically fit are described as more important for men (Grande & Romppel, 2011; Kristofferzon et al., 2003). Research has also shown that women value the emotional support and social interaction during CRP more than men and men appreciate the opportunity for information exchange more than women (Barlow, Turner, & Gilchrist, 2009). However, in more recent studies both women and men describe that they want to be supported in finding new values in life (Kazimiera Andersson, Borglin, & Willman, 2013; Pryor et al., 2014; Simony et al., 2015), which is also in line with our women's descriptions. In our study, we have not investigated differences between genders, but our result does not confirm the traditional gender-role patterns because the women also described values that seem to be more suitable for males. There is a need to further investigate the gender perspective among women and men during their recovery process following MI.

## Conclusions and implications

The women's personal recovery is a multidirectional process with a desire to develop and approach a new perspective on life. The women's possibility to approach new perspectives on life incorporates how they handle the three dimensions: behaviour, that is, women's acting and engaging in various activities; social, that is, how women receive and give support in their social environment; and psychological, that is, their way of thinking, reflecting, and appreciating life. The findings of this study suggest that CRN should focus on a recovery-oriented holistic approach where behavioural, social, and psychological dimensions are taken into account in clinical nursing and in CRP. Furthermore, studies are needed that investigate how a holistic recovery process approach including behavioural, social, and psychological dimensions can be operationalized in CRP, and how this implementation impacts on women's recovery?
